# Impact of Supine versus Prone Positioning on Segmental Lumbar Lordosis in Patients Undergoing ALIF Followed by PSF: A Comparative Study

**DOI:** 10.3390/jcm13123555

**Published:** 2024-06-18

**Authors:** Sina Sadeghzadeh, Kelly H. Yoo, Ivan Lopez, Thomas Johnstone, Ethan Schonfeld, Ghani Haider, Neelan J. Marianayagam, Martin N. Stienen, Anand Veeravagu

**Affiliations:** 1 Department of Neurosurgery, Stanford University, 453 Quarry Road, Stanford, CA 94305, USA; sinas@stanford.edu (S.S.); ivlopez@stanford.edu (I.L.); tjohnst3@stanford.edu (T.J.); eschon22@stanford.edu (E.S.); ghanih@stanford.edu (G.H.); njm39@stanford.edu (N.J.M.); 2 Department of Neurosurgery & Spine Center of Eastern Switzerland, Cantonal Hospital St. Gallen, Rorschacher Str. 95, CH-9007 St. Gallen, Switzerland; martin.stienen@kssg.ch

**Keywords:** spine surgery, spinal instrumentation, ALIF, PSF, segmental lumbar lordosis

## Abstract

**Background**: Anterior lumbar interbody fusion (ALIF) and posterior spinal fusion (PSF) play pivotal roles in restoring lumbar lordosis in spinal surgery. There is an ongoing debate between combined single-position surgery and traditional prone-position PSF for optimizing segmental lumbar lordosis. **Methods**: This retrospective study analyzed 59 patients who underwent ALIF in the supine position followed by PSF in the prone position at a single institution. Cobb angles were measured preoperatively, post-ALIF, and post-PSF using X-ray imaging. One-way repeated measures ANOVA and post-hoc analyses with Bonferroni adjustment were employed to compare mean Cobb angles at different time points. Cohen’s d effect sizes were calculated to assess the magnitude of changes. Sample size calculations were performed to ensure statistical power. **Results**: The mean segmental Cobb angle significantly increased from preoperative (32.2 ± 13.8 degrees) to post-ALIF (42.2 ± 14.3 degrees, Cohen’s d: −0.71, *p* < 0.0001) and post-PSF (43.6 ± 14.6 degrees, Cohen’s d: −0.80, *p* < 0.0001). There was no significant difference between Cobb angles after ALIF and after PSF (Cohen’s d: −0.10, *p* = 0.14). The findings remained consistent when Cobb angles were analyzed separately for single-screw and double-screw ALIF constructs. **Conclusions**: Both supine ALIF and prone PSF significantly increased segmental lumbar lordosis compared to preoperative measurements. The negligible difference between post-ALIF and post-PSF lordosis suggests that supine ALIF followed by prone PSF can be an effective approach, providing flexibility in surgical positioning without compromising lordosis improvement.

## 1. Introduction

With an aging population in the United States and rising rates of spinal pathologies, there has been an increase in the operative management of patients with spine deformity disorders. The rate of such surgeries has risen to approximately 6.5 cases per 100,000 people, representing a 3.4-fold increase from 2004 to 2011 [1]. Anterior lumbar interbody fusion (ALIF) and posterior spinal fusion (PSF) represent cornerstone surgical techniques in the comprehensive management of degenerative disc disease, lumbar lordosis restoration, and spinal fusion [2]. Despite sharing the common objective of achieving spinal stability, these procedures are distinguished by their unique approaches and positional considerations.

ALIF is typically performed using an anterior retroperitoneal approach, commonly in the supine or lateral decubitus position. This approach involves the insertion of interbody cages and the placement of bone graft material to reconstruct and stabilize the anterior column of the spine. ALIF is often favored for its direct access to the intervertebral disc space, facilitating optimal restoration of disc height and alignment. In contrast, PSF is executed through a posterior approach, predominantly with the patient positioned prone on the operating table. During PSF, pedicle screws and rods are inserted to stabilize the posterior elements of the spine, providing additional support and stability. This approach allows for comprehensive decompression of neural elements and fusion of multiple spinal levels, making it particularly suitable for addressing complex spinal pathologies. Both ALIF and PSF have demonstrated favorable outcomes in terms of achieving spinal fusion, alleviating pain, and improving functional outcomes in appropriately selected patients. However, the choice between these procedures depends on various factors, including the location and extent of spinal pathology, patient comorbidities, and surgeon expertise.

The existing literature consistently demonstrates the effectiveness of ALIF and PSF in mitigating pain, improving functional outcomes, and reducing disability in patients with spinal degenerative conditions [2,3,4,5,6]. The amalgamation of ALIF and PSF, often performed sequentially with ALIF in the supine position followed by PSF in the prone position, has gained prominence as a favored approach [7]. This dual-position strategy is recognized for its ability to enhance circumferential fusion, which is a crucial objective in managing complex spinal interventions [8].

However, the integration of ALIF and PSF techniques presents unique challenges that require careful consideration. The dual-position strategy demands meticulous orchestration to strike a balance between achieving optimal surgical outcomes and managing the complexities associated with patient repositioning [9]. There is ongoing debate within the neurosurgical community regarding the optimal patient positioning for spinal fusion surgeries. While some surgeons advocate for lateral decubitus positioning as a pragmatic alternative to mitigate the challenges associated with dual-position surgery, others argue that prone PSF may optimize segmental lumbar lordosis compared to single-position surgery [10,11].

It is also worth mentioning that complications such as rod failures, the need for revision surgeries, and the impact of patient comorbidities can influence surgical outcomes [12,13,14]. For example, Daher et al. reported that while ALIF showed slight improvements in patient-reported outcomes, the differences in complications, sagittal and coronal alignment, and overall patient-reported outcomes between ALIF and transforaminal lumbar interbody fusion (TLIF) were minimal, making TLIF the preferred approach due to the reduced comorbidities associated with the double approach required for ALIF. Even with precautionary measures in place, complications from ALIF can still occur in up to 14.5% of patients [15]. These considerations underscore the importance of conducting nuanced investigations and adopting evidence-based decision-making in spinal interventions.

In this study, we aim to investigate the impact of prone positioning on segmental lumbar lordosis subsequent to anterior column reconstruction via supine ALIF. We hypothesize that there is no significant difference in segmental lumbar lordosis between supine ALIF and prone PSF, as measured by the Cobb angle, and that both positions result in higher lordosis compared to baseline measurements. By comparing preoperative, intraoperative, and postoperative radiographic data, we seek to inform evidence-based clinical approaches, optimize patient outcomes, and drive innovative advancements within the realm of spine neurosurgery.

## 2. Methods

### 2.1. Study Design and Patient Selection

This retrospective study aimed to evaluate changes in lumbar lordosis among patients undergoing combined ALIF and PSF for degenerative lumbar diseases. The study period spanned from January 2020 to July 2023, during which time lumbar interbody fusion procedures were performed at our institution by a single surgeon. Adult patients aged 18 years and above who underwent ALIF in the supine position followed by PSF in the prone position during the same admission were included in the analysis. Exclusion criteria encompassed patients with incomplete or missing relevant imaging records and those who had undergone PSF prior to ALIF. This exclusion criterion aimed to ensure the homogeneity of the study population and minimize confounding factors related to prior surgical interventions. The retrospective design was chosen due to its ability to efficiently assess a large cohort of patients undergoing specific surgical procedures within a defined timeframe. Additionally, the single-surgeon approach ensured consistency in surgical techniques and minimized variability in surgical practices.

### 2.2. Ethical Considerations

This study involved the analysis of de-identified patient data, approved by the Institutional Review Board (IRB) at our institution (eProtocol #64120) prior to the commencement of the study. The IRB review process ensured that the study protocol adhered to ethical principles and regulatory requirements governing research involving human participants.

To protect patient confidentiality and privacy, all data were de-identified before analysis, with unique patient identifiers removed or encrypted to prevent the identification of individual participants. Additionally, strict data security measures were implemented to safeguard the confidentiality of patient information throughout the study duration. Informed consent was waived by the IRB given the retrospective nature of the study and the use of de-identified patient data. Patients were provided with information regarding the research objectives and their rights regarding the use of their medical records for research purposes in accordance with institutional policies and regulatory guidelines. The study was conducted in accordance with the principles outlined in the Declaration of Helsinki and other applicable ethical guidelines for research involving human participants.

### 2.3. Data Collection

Demographic information, surgical characteristics, and radiographic data were systematically extracted from electronic health records (EHRs) for all eligible patients. The data collection process adhered to established guidelines and protocols to ensure accuracy and consistency.

Preoperative radiographs consisted of standing lateral views, providing baseline measurements of lumbar lordosis prior to surgery. The post-ALIF radiograph was obtained in the supine position, typically while the patient was under anesthesia before repositioning for prone PSF. This imaging modality allowed for the assessment of lumbar lordosis immediately following ALIF, capturing the immediate postoperative status of the anterior column reconstruction. Subsequently, the final radiograph was acquired post-PSF, with the patient in the prone position. This radiographic view enabled the evaluation of lumbar lordosis following the completion of both ALIF and PSF procedures, providing insights into the overall postoperative spinal alignment. The timing and selection of radiographic views were carefully considered to facilitate a comprehensive analysis of lumbar lordosis changes throughout the surgical process. By capturing preoperative, intraoperative, and postoperative radiographic data, the study aimed to elucidate the dynamic changes in lumbar lordosis resulting from combined ALIF and PSF.

### 2.4. Lordosis Measurements

Cobb angles were measured using standardized techniques between the superior endplate of the uppermost instrumented vertebral body and the inferior endplate of the lowermost instrumented vertebral body. This measurement encompassed both global lumbar lordosis (from L1 to S1) and segmental lordosis at the operated level.

**Figure 1:**
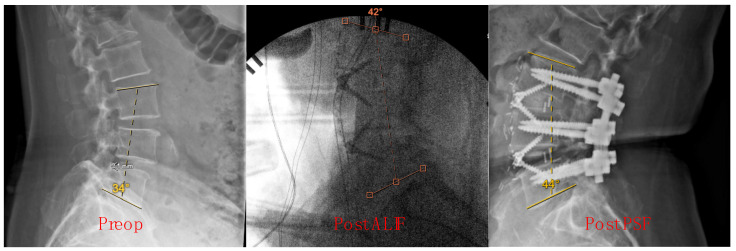
Cobb angle measurement at preop, post-ALIF, and post-PSF timepoints.

Measurements were conducted at three key time points: before ALIF in the standing position, post-ALIF in the supine position, and post-PSF in the prone position. These specific time points were chosen to capture the dynamic changes in lumbar lordosis throughout the surgical process, from preoperative baseline to immediate postoperative status. The choice of measurement technique was based on its widespread use in clinical practice and its established reliability in assessing spinal alignment [16,17,18]. Additionally, the selected measurement points were consistent with standard protocols for evaluating lumbar lordosis in spinal surgery research [19]. Sample X-ray images illustrating the calculation of preop, post-ALIF, and post-PSF Cobb angles are shown in Figure 1.

### 2.5. Statistical Analysis

The statistical analysis was conducted using the R statistical software (version 3.6.2, R Foundation for Statistical Computing). To compare within-patient means across the different time points (pre-ALIF, post-ALIF, and post-PSF), a one-way repeated measures analysis of variance (ANOVA) was employed. This statistical test was chosen to assess whether there were significant differences in lumbar lordosis measurements over the course of the surgical intervention. Post-hoc analyses were performed using Bonferroni adjustments to determine pairwise differences between the time points while controlling for multiple comparisons. This approach was used to minimize the risk of type I errors by adjusting the significance level accordingly.

Assumptions underlying the ANOVA, such as the normal distribution of residuals and homogeneity of variances, were checked to ensure the validity of the results. Additionally, potential confounding factors, such as age, gender, and diagnosis, were considered and accounted for in the analysis where appropriate. Significance was set at *p* < 0.05, indicating that differences between means were considered statistically significant if the probability of observing such differences by chance alone was less than 5%.

Sample size calculations were performed to ensure a statistical power of 80% at an alpha level of 0.05. Based on the observed effect sizes, the required sample sizes were as follows: for Preop vs. Post ALIF, 18 participants; for Post ALIF vs. Post PSF, 830 participants; and for Preop vs. Post PSF, 14 participants. With a sample size of 59 patients, the study was adequately powered to detect large effect sizes.

## 3. Results

A total of 59 patients were included, meeting the required sample size for detecting large effect sizes. The cohort had a mean age of 65 ± 10.72 years, with a male-to-female ratio of 0.78. The age range within the cohort varied from 45 to 85 years, reflecting a diverse patient population. Predominant diagnoses included spondylolisthesis (n = 48, 81.4%), spinal stenosis (n = 56, 94.9%), and disc degeneration (n = 29, 49.2%). These diagnoses were established based on clinical evaluations, imaging studies, and diagnostic criteria established by relevant professional societies. In addition, Cohen’s d effect sizes were calculated to assess the magnitude of changes in segmental lumbar lordosis at different time points. The effect size for Preop vs. Post ALIF was −0.71 (95% CI: −0.91 to −0.52), indicating a large effect size. The effect size for Post ALIF vs. Post PSF was −0.10 (95% CI: −0.23 to 0.03), indicating a negligible effect size. The effect size for Preop vs. Post PSF was −0.80 (95% CI: −0.99 to −0.61), also indicating a large effect size.

Surgical intervention typically involves the fusion of an average of 1.83 ± 0.72 levels by ALIF and 2.61 ± 1.79 levels by PSF. The choice of fusion levels was determined based on preoperative imaging findings, symptomatology, and intraoperative assessment of spinal stability. ALIF constructs varied among the cohort, with 22% (n = 13) receiving single screw constructs, 61% (n = 36) receiving two screw constructs, and 16.9% (n = 10) receiving a combination of constructs. The decision on the type of ALIF construct was individualized for each patient, taking into account factors such as vertebral anatomy, pathology, and anticipated biomechanical demands postoperatively. Detailed demographic characteristics of the patient cohort are summarized in Table 1, while Table 2 presents surgical details including fusion levels and ALIF constructs.

### 3.1. Lumbar Lordosis Measurements

**Figure 2:**
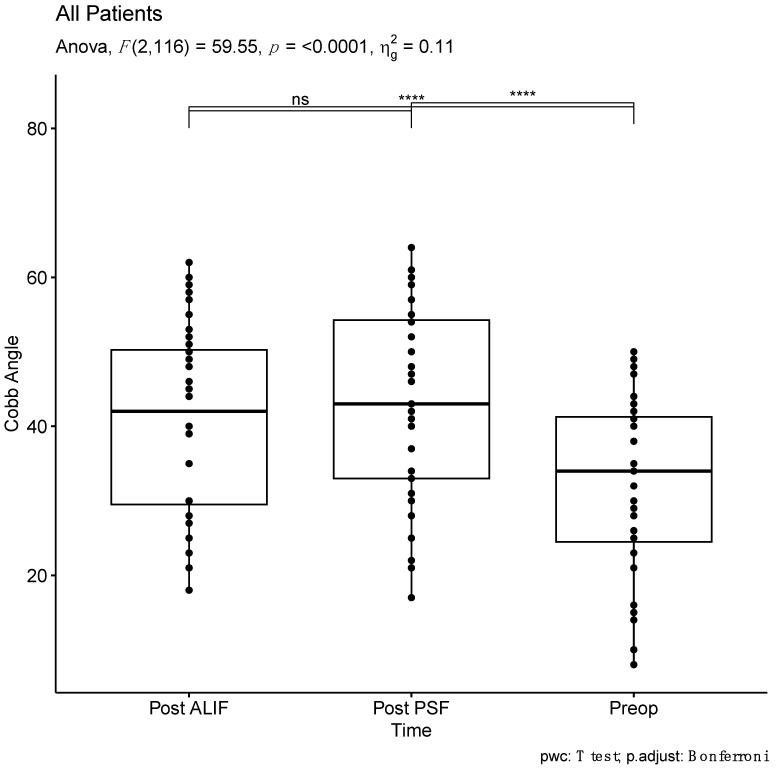
Comparative analysis of Cobb angles across all patients at three time points: pre-operative (Preop), post-anterior lumbar interbody fusion (Post ALIF), and post = posterior spinal fusion (Post PSF) **** *p* < 0.0001; ns, no significance).

The mean preoperative segmental lumbar lordosis Cobb angle was 32.2 ± 13.8 degrees, exhibiting a significant increase to 42.2 ± 14.3 degrees post-ALIF and to 43.6 ± 14.6 degrees post-PSF (Table 3). Notably, no extreme outliers were identified, and the angles demonstrated a normal distribution across all three time points (Supplemental Figure S1). Statistical analysis revealed a significant difference in Cobb angles across the observed time points (F (2, 116) = 59.55, *p* < 0.0001, generalized eta squared = 0.11). Post-hoc analyses with Bonferroni adjustment indicated no significant difference between Cobb angles after ALIF and after PSF (*p* = 0.14). Moreover, both post-ALIF and post-PSF segmental lumbar lordosis were significantly higher than the preoperative measurements (*p* < 0.0001 for both) (Table 3, Figure 2).

### 3.2. Subgroup Analysis

**Figure 3:**
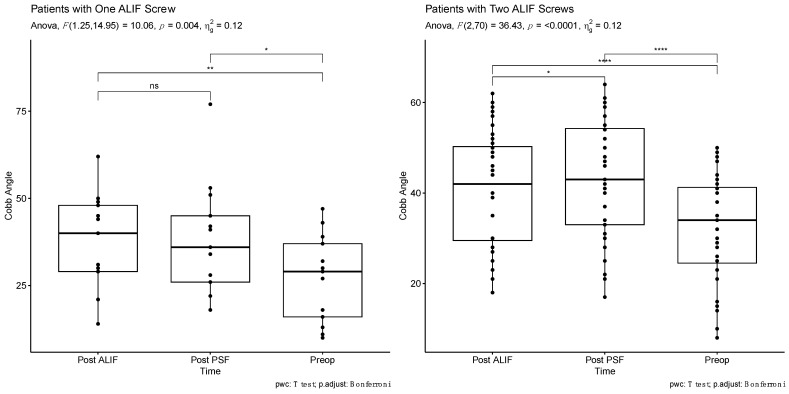
Cobb angle comparison based on alif construct type at three time points: pre-operative (Preop), post-anterior lumbar interbody fusion (Post ALIF), and post-posterior spinal fusion (Post PSF) (Single Screw vs. Double Screw) (* *p* < 0.05, ** *p* < 0.01, **** *p* < 0.0001; ns, no significance).

In patients with two-screw ALIF constructs, segmental lumbar lordosis demonstrated a significant increase post-ALIF (40.5 ± 12.7 degrees) and post-PSF (43.5 ± 13.1 degrees) compared to preoperative measurements (32.4 ± 12.3 degrees) (*p* < 0.0001 for both) (Table 3). Notably, post-PSF segmental lumbar lordosis was significantly higher than post-ALIF segmental lumbar lordosis (*p* = 0.043). Conversely, in the single-screw ALIF cohort, segmental lumbar lordosis increased significantly post-ALIF (37.2 ± 14.1 degrees) and post-PSF (38.1 ± 16.2 degrees) compared to preoperative values (27.1 ± 12.5 degrees) (*p* = 0.003 and *p* = 0.031, respectively). However, no significant difference was observed between post-ALIF and post-PSF segmental lumbar lordosis (*p* = 1) (Table 3, Figure 3).

Importantly, it is worth noting that while statistically significant changes in segmental lumbar lordosis were observed following both ALIF and PSF procedures, the clinical significance of these changes in terms of functional outcomes and patient satisfaction warrants further investigation. Additionally, the absence of significant demographic differences between groups and the normal distribution of angles at all time points (Supplemental Figure S1) support the robustness of the findings.

## 4. Discussion

Our study delved into the intricate dynamics of lumbar spine surgery, with a particular focus on exploring positional variations during ALIF followed by PSF. The objective was to unravel the nuances of segmental lumbar lordosis enhancement through the sequential use of supine ALIF and prone PSF techniques. Our findings support the hypothesis that both supine ALIF and prone PSF result in significant increases in segmental lumbar lordosis compared to preoperative measurements with equivalent outcomes between the two positions. These findings suggest flexibility in surgical positioning without compromising the outcome of lumbar lordosis improvement.

Historically, prone positioning has been conventionally favored in spinal surgeries, particularly in the context of PSF, with the belief that it optimally restores segmental lumbar lordosis [20,21]. However, emerging evidence and clinical experiences have prompted a reevaluation of this longstanding practice. Our analysis challenges the traditional notion surrounding prone positioning in lumbar spine surgeries and seeks to provide empirical insights into the efficacy of alternative approaches, such as the combination of supine ALIF followed by prone PSF.

### 4.1. Comparative Analysis: Supine ALIF versus Prone PSF

In contrast to prevailing notions, our study reveals that both supine ALIF and prone PSF result in comparable and significantly higher increases in segmental lumbar lordosis compared to preoperative measurements. This observation challenges the orthodoxy surrounding the necessity of prone positioning for achieving superior lordotic outcomes in lumbar spine surgery [2,22]. The comparable outcomes between supine ALIF and prone PSF suggest a potential paradigm shift in surgical approaches, offering avenues for more streamlined procedures that can reduce operative time, anesthesia requirements, and overall resource utilization.

These findings carry significant implications for clinical practice, suggesting that surgeons may have more flexibility in selecting the optimal approach for lumbar spine surgery based on patient-specific factors and surgical preferences. In addition, the potential reduction in resource utilization associated with supine ALIF may have implications for healthcare delivery and cost-effectiveness. Further research and prospective studies are warranted to validate these findings and explore their long-term impact on patient outcomes and healthcare resource allocation.

### 4.2. Significance of ALIF Constructs

Our investigation has yielded significant insights into the influence of different ALIF constructs on lordosis enhancement. Specifically, employing two-screw ALIF configurations led to statistically significant increases in lordosis measurements, highlighting their efficacy in achieving favorable outcomes compared to one-screw ALIF constructs. The observed consistency in lordosis levels post-ALIF and post-PSF within the one-screw ALIF group aligns with broader trends observed in the literature [23].

This nuanced understanding underscores the pivotal role of tailored ALIF constructs customized to individual patient anatomy and pathology. By discerning the varying impact of different constructs on lordosis, clinicians can navigate surgical decision-making more effectively to optimize patient outcomes. Moreover, these findings emphasize the importance of adopting a personalized approach in spinal surgery, where treatment strategies are meticulously adjusted to each patient’s unique characteristics.

Furthermore, these findings have implications for surgical planning and patient management. Surgeons may consider the use of two-screw ALIF constructs in cases where greater lordosis enhancement is desired while recognizing that one-screw ALIF constructs may provide satisfactory outcomes in select patients. Additionally, further research exploring the biomechanical mechanisms underlying the observed differences in lordosis enhancement between diverse ALIF constructs could provide valuable insights into optimizing surgical techniques and improving patient outcomes.

### 4.3. Clinical Implications and Resource Optimization

Our findings carry significant clinical implications, particularly in the realms of resource optimization and patient safety. Single-position surgeries, as demonstrated in our research, offer clear advantages, including reduced operative duration, decreased anesthesia requirements, and minimized risks associated with patient repositioning. Prolonged repositioning periods have been associated with increased susceptibility to infection, blood loss, and other perioperative complications [24]. Furthermore, prone positioning presents inherent risks, encompassing postoperative vision impairment, cardiovascular events, hypovolemia, compromised pulmonary compliance, and potential cardiac arrest [25].

While prone positioning has traditionally been favored in spinal surgeries, our study advocates for the adoption of more efficient and streamlined surgical approaches, such as supine ALIF. By highlighting the comparable effectiveness of supine ALIF, our findings support a paradigm shift towards single-position procedures, which not only improve operative efficiency but also enhance patient safety and foster better postoperative outcomes.

However, it is important to acknowledge potential challenges associated with the adoption of supine ALIF, such as access to the surgical site or technical feasibility in certain cases. Additionally, double approach–related comorbidities are a concern with ALIF. A comparative meta-analysis of ALIF with TLIF by Daher et al. found no significant difference in complications, sagittal and coronal alignment, and patient-reported outcomes between ALIF and TLIF, favoring TLIF for adult spinal deformity surgery [26]. Surgeons should carefully consider the balance between reduced complications and potential drawbacks when evaluating the suitability of supine ALIF as a standalone strategy, taking into account patient-specific factors and clinical considerations.

### 4.4. Validity of Supine Lumbar Lordosis Calculation and Comparisons to Standing Radiographs

While interpreting these findings, it is crucial to note that segmental lumbar lordosis measurements in supine and prone positions may not directly compare to those calculated from pre-operative standing radiographs. For instance, in a retrospective cohort study of 56 patients with sagittal malalignment, Fourman et al. found that supine positioning improved distal lumbar lordosis but did not fully correct to age-matched norms, particularly in the proximal lumbar spine, indicating that gravity significantly influences lordosis distribution in patients with sagittal malalignment [27]. Furthermore, in a retrospective study of 63 patients, Benditz et al. noted that standing radiographs typically showed slightly higher lordosis values compared to supine Magnetic Resonance Images (MRIs), especially at L5/S1, indicating that MRI can estimate global lumbar lordosis, but single-level lordosis should ideally be determined using standing radiographs [28]. Similarly, in a retrospective study of 82 patients undergoing lumbar fusion, Greimel et al. found that intraoperative lordosis angles closely matched the postoperative measurements from standing radiographs, particularly at the L4-S1 level, despite prone positioning during the surgery [29]. Lastly, in a single-center retrospective study of 91 adult spinal deformity (ASD) patients, Lovecchio et al. found that patients who did not undergo osteotomy had supine alignment similar to postoperative alignment, while those who did undergo osteotomies showed differences in alignment between preoperative standing, preoperative supine, and postoperative standing lumbar curvatures [30]. These findings suggest that while supine and prone imaging provide valuable intraoperative and immediate postoperative data, they may not fully represent true sagittal alignment, and surgeons should consider these discrepancies when planning surgeries and interpreting results.

### 4.5. Limitations

While our study provides valuable insights, it is imperative to acknowledge its limitations. The retrospective design inherently introduces selection bias, compounded by the absence of a control group, which may have influenced the observed outcomes. Additionally, the single-center, single-surgeon approach employed in our study may limit the generalizability of our findings to broader patient populations, as variations in surgical techniques and patient demographics across different centers may yield different results.

Furthermore, the lack of postoperative follow-up data impedes the accurate assessment of long-term outcomes, potentially limiting the comprehensive understanding of the efficacy and durability of the surgical interventions studied. It is noteworthy that the emerging literature challenges the conventional requirement of prone positioning for achieving optimal lordotic outcomes, advocating alternative techniques such as XLIF, OLIF, or supine ALIF. The absence of postoperative follow-up data may have precluded the opportunity to evaluate the comparative effectiveness of these alternative techniques, which could have implications for the interpretation of our results.

Moreover, the extent of lordosis improvement observed in our study is intricately influenced by various factors, including the surgical level, prior interventions, bone quality, and utilization of osteotomies. While our study aimed to control for these factors to the extent possible, residual confounding may still exist, which should be considered when interpreting the results.

Moving forward, future research efforts must address these limitations by encompassing larger, more diverse patient cohorts, employing standardized methodologies, and incorporating long-term follow-up evaluations. Such endeavors are indispensable for validating our findings and establishing comprehensive guidelines to guide optimal surgical decision-making in spinal procedures.

## 5. Conclusions

In conclusion, our study challenges the traditional preference for prone positioning in posterior spine surgeries by demonstrating the comparable efficacy of supine ALIF followed by prone PSF in enhancing segmental lumbar lordosis. These findings suggest that a dual-position strategy may not provide additional clinical benefits and may complicate procedures with longer operative times, increased anesthesia requirements, and associated risks compared to single-position approaches. Moreover, embracing evidence-based practices and innovative techniques is essential for driving the evolution of lumbar spine surgeries, ultimately leading to improved patient outcomes and optimized resource utilization in neurosurgical interventions in spine neurosurgery.

## Figures and Tables

**Table 1:** Patient demographics.

	Overall (N = 59)
**Sex**	
F	33 (55.9%)
M	26 (44.1%)
**Age**	
Mean (SD)	65.9 (10.7)
Median [Min, Max]	67.0 [42.0, 87.0]
**Spondylolisthesis**	
Yes	48 (81.4%)
No	11 (18.6%)
**Spinal Stenosis**	
Yes	56 (94.9%)
No	3 (5.1%)
**Disc Degeneration**	
Yes	29 (49.2%)
No	30 (50.8%)
**Coronary Artery Disease**	
Yes	13 (22.0%)
No	46 (78.0%)
**Osteoporosis**	
Yes	8 (13.6%)
No	51 (86.4%)
**Type II Diabetes Mellitus**	
Yes	6 (10.2%)
No	53 (89.8%)
**Tobacco Use Disorder**	
Yes	12 (20.3%)
No	47 (79.7%)
**BMI**	
Mean (SD)	29.6 (5.62)
Median [Min, Max]	28.6 [19.0, 40.7]

*F, Female; M, Male; BMI, Body mass index; N, Number; SD, Standard deviation; Min, Minimum, Max, Maximum.*

**Table 2:** Surgical characteristics.

	Overall (N = 59)
**No. Fused Vertebrea—ALIF**	
Mean (SD)	1.83 (0.723)
Median [Min, Max]	2.00 [1.00, 3.00]
**No. Fused Vertebrea—PSF**	
Mean (SD)	2.61 (1.79)
Median [Min, Max]	2.00 [1.00, 9.00]
**No. ALIF Screws**	
1	13 (22.0%)
2	36 (61.0%)
Both	10 (16.9%)

*ALIF, Anterior lumbar interbody fusion; PSF, Posterior spinal fusion N, Number; SD, Standard deviation; Min, Minimum, Max, Maximum.*

**Table 3:** Segmental Cobb angle measurements and effect sizes.

Cohort	Time Point	Segmental Cobb Angle Mean (SD)	Cohen’s d (95% CI)	*p*-Value
Whole Cohort	Preoperative	32.2 (13.8)	REF	REF
	Post ALIF	42.2 (14.3)	−0.71 (−0.91, −0.52)	*p < 0.0001*
	Post PSF	43.6 (14.6)	−0.80 (−0.99, −0.61)	*p < 0.0001*
	Post ALIF vs. Post PSF	NA	−0.10 (−0.22, 0.03)	*p* = 0.14
One-screw ALIF	Preoperative	27.1 ± 12.5	REF	REF
	Post ALIF	37.2 ± 14.1	−0.75 (−1.16, −0.34)	*p = 0.003*
	Post PSF	38.1 ± 16.2	−0.73 (−1.30, −0.17)	*p = 0.031*
	Post ALIF vs. Post PSF	NA	−0.05 (−0.30, 0.19)	*p* = 1
Two-screw ALIF	Preoperative	32.4 (12.3)	REF	REF
	Post ALIF	40.5 (12.7)	−0.64 (−0.91, −0.38)	*p < 0.0001*
	Post PSF	43.5 (13.1)	−0.87 (−1.12, −0.62)	*p < 0.0001*
	Post ALIF vs. Post PSF	NA	−0.23 (−0.41, −0.05)	*p = 0.043*

*SD, Standard deviation; 95% CI, 95% Confidence interval; REF, Reference; NA, Not Applicable.*

## Data Availability

The data presented in this study are available upon request from the corresponding author. They are not publicly available due to patient privacy concerns under HIPAA regulations.

## Supplementary Materials

The following supporting information can be downloaded at: https://www.mdpi.com/article/10.3390/jcm13123555/s1, Figure S1: QQ plots comparing ALIF construct types (single screw, double screw, all).
