# Supplementary figures and images for: Impact of Supine versus Prone Positioning on Segmental Lumbar Lordosis in Patients Undergoing ALIF Followed by PSF: A Comparative Study
Source: J Clin Med. 2024 Jun 18;13(12):3555. doi: 10.3390/jcm13123555 (PMC11204788; doi:10.3390/jcm13123555)

Supplemental Figure S1: QQ Plots Comparing ALIF Construct Types (Single Screw, Double Screw, All)

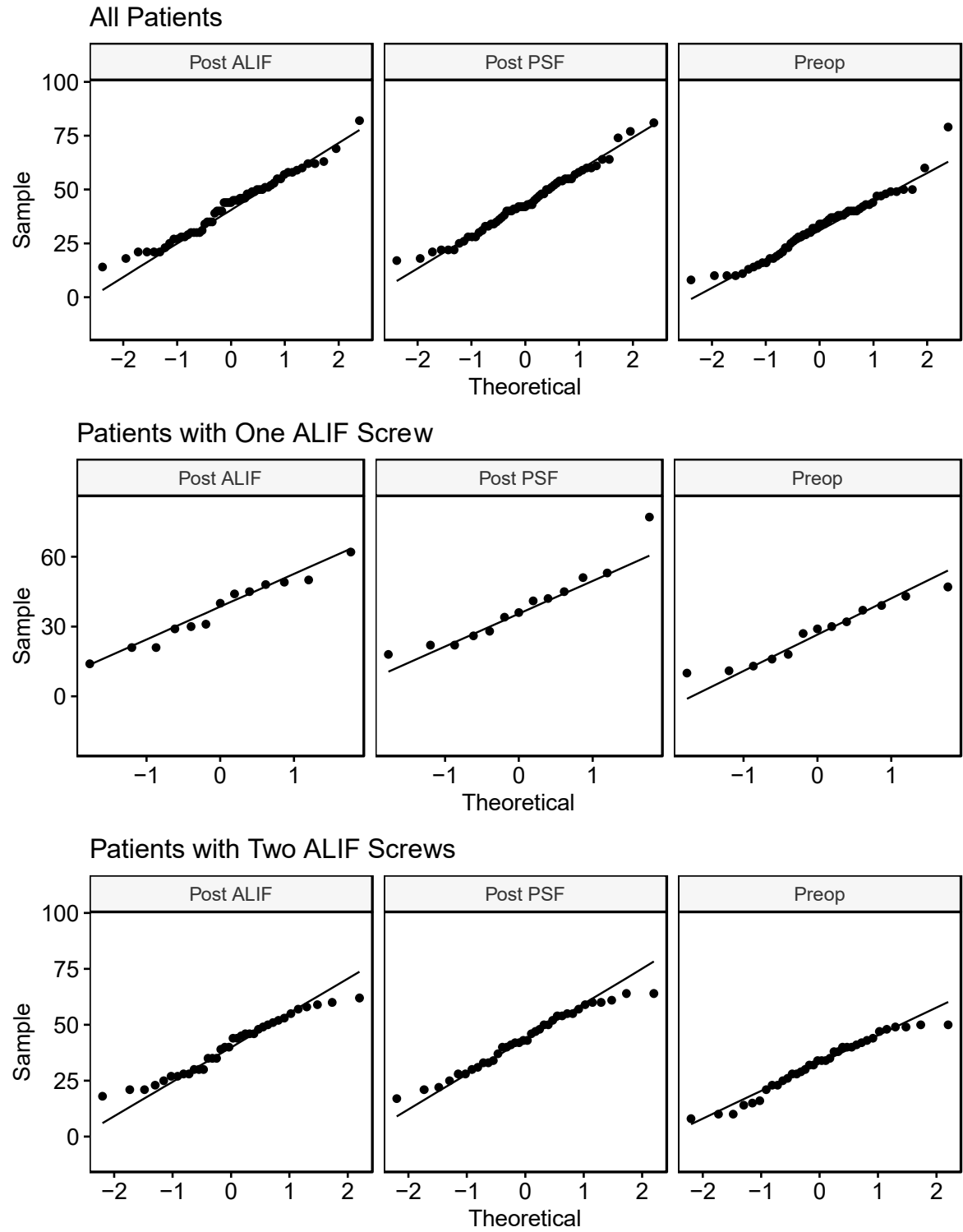

Supplement: Supplementary file 1 [file jcm-13-03555-s001.zip › jcm-2941667-supplementary.pdf]
